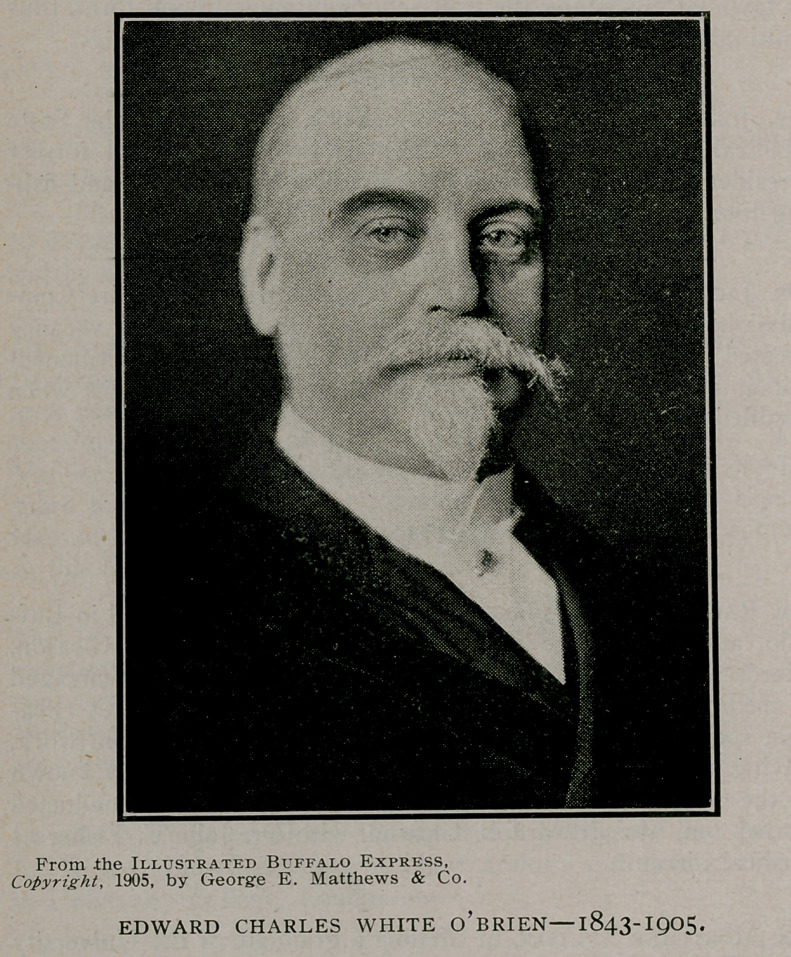# Dr. Edward Charles White O’Brien

**Published:** 1905-04

**Authors:** 


Dr. Edward Charles White O'Brien died at his residence in Buffalo, February 26, 1905, aged 62 years. A memorial, giving

From the Illustrated Buffalo Express,
*Copyright*, 1905, by George E. Matthews & Co.

EDWARD CHARLES WHITE O'BRIEN—1843-1905.

a sketch of his life, appears elsewhere in this issue under proceedings of the Medical Society of the County of Erie. His funeral was held at Bishop Colton's chapel, Delaware avenue, Wednesday, March 1, 1905, the interment being at Forest Lawn. The active bearers were: Drs. John Parmenter, Marshall Clinton, Edward M. Dooley, Arthur W. Hurd, John B. Coakley, James W. Putnam, Frank W. McGuire, and Alvin A. Hubbell.

The fire commissioners, the chief and assistant chief of the fire department, together with a detail of fifty uniformed firemen, attended the funeral. The flags on all the fire houses remained at half mast until after the funeral, Dr. O'Brien having been for many years surgeon of the fire department.

## Figures and Tables

**Figure f1:**